# LPS from *P. gingivalis* and Hypoxia Increases Oxidative Stress in Periodontal Ligament Fibroblasts and Contributes to Periodontitis

**DOI:** 10.1155/2014/986264

**Published:** 2014-10-13

**Authors:** L. Gölz, S. Memmert, B. Rath-Deschner, A. Jäger, T. Appel, G. Baumgarten, W. Götz, S. Frede

**Affiliations:** ^1^Department of Orthodontics, Dental Clinic, University Hospital of Bonn, Welschnonnenstraße. 17, 53111 Bonn, Germany; ^2^Center of Dento-Maxillo-Facial Medicine, Faculty of Medicine, University of Bonn, Welschnonnenstraße 17, 53111 Bonn, Germany; ^3^Clinic of Anesthesiology and Intensive Care Medicine, University Hospital of Bonn, Sigmund-Freud-Straße 25, 53105 Bonn, Germany

## Abstract

Oxidative stress is characterized by an accumulation of reactive oxygen species (ROS) and plays a key role in the progression of inflammatory diseases. We hypothesize that hypoxic and inflammatory events induce oxidative stress in the periodontal ligament (PDL) by activating NOX4. Human primary PDL fibroblasts were stimulated with lipopolysaccharide from *Porphyromonas gingivalis* (LPS-PG), a periodontal pathogen bacterium under normoxic and hypoxic conditions. By quantitative PCR, immunoblot, immunostaining, and a specific ROS assay we determined the amount of NOX4, ROS, and several redox systems. Healthy and inflamed periodontal tissues were collected to evaluate NOX4 and redox systems by immunohistochemistry. We found significantly increased NOX4 levels after hypoxic or inflammatory stimulation in PDL cells (*P* < 0.001) which was even more pronounced after combination of the stimuli. This was accompanied by a significant upregulation of ROS and catalase (*P* < 0.001). However, prolonged incubation with both stimuli induced a reduction of catalase indicating a collapse of the protective machinery favoring ROS increase and the progression of inflammatory oral diseases. Analysis of inflamed tissues confirmed our hypothesis. In conclusion, we demonstrated that the interplay of NOX4 and redox systems is crucial for ROS formation which plays a pivotal role during oral diseases.

## 1. Introduction

Oxidative stress describes a metabolic state where the amount of reactive oxygen species (ROS) is strikingly increased above physiological levels. ROS originate from the incomplete reduction of molecular oxygen resulting in the accumulation of oxidants like hydrogen peroxide (H_2_O_2_) or free radicals like the hyperoxide (superoxide) anion (O_2_^∙−^). They are continuously generated in cells as byproducts of normal aerobic metabolic pathways through the oxidative phosphorylation chain. Extrinsic factors like radiation, hypoxia, smoking, heat, trauma, chemotherapy, growth factors, or cytokines may augment ROS formation [1–3]. Low or physiological levels of ROS can activate intracellular signaling pathways related to proliferation, differentiation, and other cellular functions, whereas uncontrolled release may damage DNA, lipids, or proteins potentially leading to mutagenesis or carcinogenesis [4–7] as many cancer cells were shown to have increased levels of ROS. Thereby, ROS are promoting the invasion and progression of tumors via matrix metalloproteinase (MMP) activation.

The primary ROS, O_2_^∙−^, is mainly produced by monoamine oxidases, so-called NADPH-oxidase (NOX) proteins, at complexes I and III of the electron transport chain [8]. NOX proteins are expressed in a variety of tissues and represent the only known enzyme system whose primary biological function is to produce ROS. They catalyze the reduction of oxygen to superoxide using NADPH as an electron donor [9]. These enzymes were first described in phagocytes where the respiratory burst works as an important defense mechanism against bacteria. In the last decades five NOX enzymes (NOX 1-5) and two dual oxidases (Duox), Duox1 and Duox2, have been discovered which were shown to be expressed not only in phagocytes, but also in epithelial cells, adipocytes, or cardiac fibroblasts [9, 10].

The NADPH-oxidase family member NOX4, originally Renox, was first described in kidney cells where it is highly expressed [11]. Later on, NOX4 was also detected in other cell types such as endothelial cells, fibroblasts, keratinocytes, and osteoclasts [9, 11, 12]. The typically transmembranously located enzyme is upregulated in response to endoplasmatic reticulum stress [13], shear stress [14], hypoxia, or ischemia [15, 16]. In addition, cytokines like transforming growth factor- (TGF-) *β*1, tumor necrosis factor- (TNF-)*α* and insulin like growth factor- (IGF-) 1 induce NOX4 expression [9, 17]. Furthermore, this enzyme seems to be a part of the cellular oxygen sensing mechanisms. This is supported by the fact that multiple interactions exist between NOX4, hypoxia inducible factor-1 alpha (HIF-1*α*), and ROS [18, 19]. NOX4-derived ROS have been associated with a variety of processes, for example, apoptosis, insulin signaling, cell differentiation and senescence, and bone remodeling [9, 20, 21].

To limit the potentially deleterious effects of ROS several protective mechanisms have been evolved. In mitochondria, metabolic uncoupling is one mechanism to regulate ROS production. In healthy cells, excessive ROS are decomposed by a protective antioxidant machinery. According to their catalyzing function in the ROS detoxification process, these redox enzymes are grouped in different systems or families, like the superoxide dismutase (SOD), catalase (CAT), the glutathione system, or thioredoxin. Furthermore, there are also nonenzymatic or small molecules including vitamins (C and E), pyruvate, flavonoids, ubiquinone, carotinoids, and many plant-derived antioxidants involved in ROS scavenging [6].

Oxidative stress is gaining increasing interest as an important cofactor in the etiology and pathogenesis of different oral and dental diseases including pulpal or mucosal inflammation, tumor development, or inflammatory processes like periodontitis. First evidence for the presence and role of ROS in the periodontium was given by the detection of these molecules after respiratory burst by polymorphonuclear lymphocytes (PMNLs) in periodontal damage [22]. Since then, the implication of ROS and their degrading enzymes SOD or CAT in the pathogenesis of periodontal diseases has been supported by several groups [23–26]. High ROS levels may also be a linking element between obesity, diabetes mellitus, atherosclerosis, and chronic periodontitis [27]. In addition, there are many intervention studies investigating antioxidants and antioxidant micronutrients in periodontal therapy including the use of mouth rinses and toothpastes. Some of these studies provided evidence for beneficial effects, but the links between certain antioxidant species and pathogenic mechanisms are still missing [22]. This is also the case for the appearance of ROS and redox systems in the healthy human oral cavity or periodontium.

The periodontium consists of the alveolar bone, the tooth, and the gingiva as well as the periodontal ligament (PDL) which connects the tooth and the surrounding alveolar bone. The collagenous structure of the PDL functions as a shock-reabsorbing barrier protecting the tissues against mechanical forces [28]. These forces may induce local hypoxia as well as inflammation [29]. Studies indicate that periodontal pathogenic bacteria like* Porphyromonas gingivalis* (*P. gingivalis*) trigger the onset and duration of chronic periodontitis [30].* P. gingivalis* is a gram-negative anaerobic bacterium belonging to the red complex of oral microflora in accordance with their pathogenicity [31].* P. gingivalis* is the most prominent periodontal pathogen and is known to be associated with artherosclerosis and cardiovascular diseases. To mimic the microenvironment of the gingival sulcus, which is characterized by low oxygen tension [32], we include hypoxic conditions in our experiments. Since* P. gingivalis* is an anaerobic bacterium, the hypoxic gingival sulci represent an ideal place for the proliferation of these bacteria. Moreover, inflammation by itself may contribute to the development of tissue hypoxia due to an increase in oxygen consumption, for example, by invading immune cells [33].

Resident cells in the periodontium are also involved in inflammatory and immune processes leading to cytokine (i.e., interleukin- (IL-) 1*β* or TNF-*α*) or chemokine release which could further enhance inflammation [34]. PDL fibroblasts are responsible for the maintenance, remodeling, and repair of the extracellular matrix of the PDL. Their immune modulatory capacities were demonstrated in some recent studies [35, 36]. Local ROS accumulation may promote the induction of proinflammatory cytokines with consecutive activation of macrophages [37] finally leading to periodontal destruction via MMPs [38].

*In vitro* studies evaluating oxidative stress at the cellular level have been focused on gingival fibroblasts, while PDL cells have rarely been analyzed. Under low zinc environment, human PDL fibroblasts showed increased oxidative stress [39]. Similar results were found after nicotine, ethanol, or metal exposure which was abolished after different antioxidant treatment [40, 41]. Stimulation experiments in human gingival fibroblasts (HGFs) support our hypothesis that PDL cells might play a pivotal role during periodontal diseases. The authors demonstrated that exposure to lipopolysaccharide from* P. gingivalis* (LPS-PG) or LPS from* Escherichia coli* (LPS-*E. coli*) increased superoxide concentrations [26], the levels and expression of ROS [42], and proinflammatory cytokines in HGFs [43]. ROS activation was associated with activation of the redox-sensitive transcription factor nuclear factor-kappaB (NF-*κ*B) [43], which is critically involved in the regulation of cytokine and MMP expression. The importance of NF-*κ*B was also demonstrated for the interaction between NOX4, HIF-1*α*, and ROS [18, 19].

However, evidence for the involvement of PDL cells in bacterial- or hypoxia-induced ROS formation is missing. The same is true for the balance between NOX4 and redox systems in the periodontium. The objective of the present study was therefore to define the role of NOX4 and ROS formation in the PDL especially during inflammatory and hypoxic events. We hypothesized that ROS are regulated by hypoxia and inflammatory stimuli in PDL cells and periodontal tissues due to an imbalance of NOX4 and redox system activation. The current study will enhance the knowledge of the role played by oxidative stress in periodontal tissues which might improve diagnostic and therapeutic strategies in oral diseases.

## 2. Materials and Methods

### 2.1. Isolation and Characterization of PDL Cells

Human periodontal ligament fibroblasts were harvested from caries-free and periodontally healthy premolars from patients aged 12 to 17 years (*n* = 9), which had to be extracted due to orthodontic reasons. Exclusion criteria were acute or chronic inflammation. Patients have had no medication; they received local anesthesia during extraction and analgesia after extraction. The approval of the Ethics Committee of the University of Bonn as well as parental and patients allowance was obtained. Cells were harvested as previously described [44]. In brief, cells were collected from the middle part of the tooth root to avoid contamination by cells derived from the gingiva and dental germ. Using explant technique, PDL cells were cultured in Dulbecco's minimal essential medium (DMEM, Invitrogen, Karlsruhe, Germany) supplemented with 10% fetal bovine serum (FBS, Invitrogen), 100 units of penicillin, and 100 mg/mL streptomycin (Biochrom, Berlin, Germany) at 37°C in a humidified atmosphere of 5% CO_2_. Cells from nine different individuals were phenotyped by the analysis of the known PDL markers (alkaline phosphatase (ALP), osteocalcin (OC), osteopontin (OP), periostin, and S100 A4) at 3rd passage [40]. After phenotyping three cell lines were pooled and used for experiments at 4th or 5th passage.

### 2.2. Cell Experiments

Cells were seeded (50.000 cells/well) on six-well plates and grown to 80% confluence. Cells were cultured under normoxic (21% O_2_ and 5% CO_2_) or hypoxic conditions (1% O_2_ and 5% CO_2_, INCO 153, O_2_/CO_2_ incubator; Memmert GmbH, Schwabach, Germany) for 2, 4, 8, 24, and 48 hours with or without the addition of 1 *μ*g/mL ultrapure lipopolysaccharide from* Porphyromonas gingivalis* (LPS-PG) (Invivogen, San Diego, USA). Prior to the here-presented study, we analyzed the dose-response of PDL cells with respect to the inducibility of NOX4 and H_2_O_2_ release by LPS-PG treatment. The maximal induction was detected after stimulation with 1 *μ*g/mL LPS-PG and no further increase was observed when the LPS-PG concentration was raised.

### 2.3. Analysis of Gene Expression

Cells were lysed with 4 M guanidinthiocyanat (Roth, Karlsruhe, Germany) and total RNA was extracted using the acid guanidinium thiocyanate/phenol/chloroform extraction method [45]. A total of 1 *μ*g of RNA was reverse-transcribed into cDNA by the use of 200 U of Moloney murine leukemia virus (MMLV) reverse transcriptase (Bio-Rad, Munich, Germany). cDNA expression was detected by real-time PCR (RT-PCR) using specific primers (18S: fwd: CGGCTACCACATCCAAGGAA, rev: GCTGGAATTACCGCGGCT and NOX4: fwd: TCTGCCTGTTCATCTGGCTCTCCA, rev: AGCCAAGAGTGTTCGGCACATGGGTA) and SYBR Green as fluorescent dye on the ViiA7 detection system (Applied Biosystems, Darmstadt, Germany). Two *μ*L of cDNA served as template in a 20 *μ*L reaction mixture containing 10 *μ*L QuantiTect SYBR Green Master Mix (Qiagen, Hilden, Germany). The mixture was heated initially to 50°C for 2 min and to 95°C for 15 min followed by 40 cycles with denaturation at 95°C for 15 s and annealing and extension at 60°C for 1 min. Amounts of specific cDNA were normalized to the housekeeping gene 18S and cDNA expression was calculated as relative expression of the respective control.

### 2.4. Immunoblot

For protein quantification, the Pierce BCA Protein Assay Kit (Thermo Scientific, Rockford, USA) was used. After addition of 4x sample buffer (50 mM Tris, pH 6.8, 2% (w/v) SDS, 5% (v/v) 2-mercaptoethanol, 0.0125% bromophenol blue, and 1% glycine), 50 *μ*g of total cell lysate per lane was subjected to 7.5% SDS-PAGE and transferred onto a nitrocellulose membrane (0.2 *μ*m pore size; Schleicher & Schuell Microscience, Dassel, Germany). The efficiency of protein transfer and equal loading was confirmed by staining the membrane with Ponceau S (Sigma Aldrich, Munich, Germany). Membranes were blocked overnight at 4°C with 5% (w/v) nonfat milk in TBS-T (10 mM Tris, pH 7.5, 100 mM NaCl, and 0.05% Tween-20) and washed with TBS-T prior to incubation with a rabbit polyclonal antibody against NOX4 (Abcam, Cambridge, UK) at room temperature for 2 h. ß-Actin (Santa Cruz Biotechnology, Santa Cruz, USA) served as marker for equal protein loading. After washing with TBS-T, HRP- (horseradish peroxidase-) conjugated anti-rabbit IgG antibody (New England Biolabs) was added. Immunoreactive proteins were visualized by chemiluminescence (Amersham Chemiluminescence Kit) and exposure to X-ray films (Agfa, Mortsel, Belgium). Densitometrical analysis was performed using the freely available image-processing software ImageJ 1.43 (http://rsb.info.nih.gov/ij/).

### 2.5. Amplex Red Assay

For hydrogen peroxide (H_2_O_2_) detection, the commercially available* N*-acetyl-3,7-dihydroxyphenoxazine  (Amplex  Red)  Hydrogen Peroxide/peroxidase Assay Kit (Molecular Probes, Eugene, OR) was used. The colorless reagent reacts with hydrogen peroxide (H_2_O_2_) using horseradish peroxidase (HRP) to catalyze the H_2_O_2_-dependent oxidation of nonfluorescent Amplex Red to fluorescent resorufin red. This assay detects only the release of hydrogen peroxide, since the size of HRP prevents it from entering the mitochondria. Therefore, PDL cells were incubated with 500 *μ*L Amplex Red and 0.1 U/mL peroxidase was added at 37°C for 30 min. Absorbance was measured at 565 nm after 1, 2, and 4 hours. During the procedure, excitation light intensity was kept to an absolute minimum as recommended by Zhao et al. [46].

### 2.6. Immunofluorescence

For this purpose, PDL cells were cultured on cover slips to 60% confluence. PDL cells were stained for NOX4 and redox systems under normoxic or hypoxic conditions in the presence and absence of LPS-PG. Hypoxic conditions were obtained by placing the cells in an incubator where the oxygen concentration can be adjusted to 1% O_2_, balanced with 5% CO_2_ and N_2_ in the incubation atmosphere (INCO 153, O_2_/CO_2_ incubator; Memmert GmbH, Schwabach, Germany). At the end of the experiment, medium was removed; cells were washed three times with PBS and fixed with 250 *μ*L cooled methanol/aceton (1 : 1) at −20°C for 10 min. The solution was removed and cells remained at room temperature for 10 min to dry. After blocking with 3% BSA-PBS for 10 min, cells were incubated with antibodies (Ab) directed against NOX4 (Abcam), CAT, or SOD (Abcam) 1 : 50 in PBS, 3% BSA, for 2 hours. Cells were washed with PBS before the species-specific secondary Ab (dilution 1 : 400 in PBS) were added for additional 90 min. Cells were covered on slides with DAPI containing mounting medium (Dako). On the following day cells were scanned with the Zeiss Axio Imager A1 fluorescence microscope (HBO 100; Carl, Zeiss, Jena, Germany), an AxioCam MRc camera (Carl, Zeiss), and the AxioVision 4.7 software (Carl, Zeiss). Density analysis was performed using the freely available image-processing software ImageJ 1.43 (http://rsb.info.nih.gov/ij/). Quantification of relative fluorescence intensity was performed by measuring the intensity of each cell related to cell area. To obtain grayscale pictures, each was processed using the tools “color” and “split channels” in ImageJ which resulted in red, green, and blue color-fractionated grayscale pictures for each channel. Only green fractionated pictures were used for calculation of the relative fluorescence intensity.

### 2.7. Immunohistochemistry

Periodontal tissues from clinically healthy patients (I) as well as those from patients with the diagnosis of gingivitis (II) and periodontitis (III) were obtained from three different patients for each group. Diagnoses of gingivitis and periodontitis were performed by specialists from the Clinic of Dental Medicine. Gingivitis was characterized by a reversible local inflammatory reaction of the gingiva, visible as redness, swelling, and bleeding. In contrast, periodontitis affects four different structures: gingiva, periodontal ligament, tooth, and alveolar bone. Therefore, periodontitis was diagnosed by an increase of probing depth, bleeding, and attachment loss which was supported by radiographs. Tissues were fixed in 4% phosphate-buffered paraformaldehyde for 24 h, followed by hydration and dehydration in an ascending ethanol series which ended in paraffin embedding. Afterwards, 4 *μ*m thick paraffin-embedded sections were prepared, deparaffinized, rehydrated, rinsed with TBS, pH 7.4 for 10 min, and then soaked in 70 mL methanol containing 700 mL of 30% H_2_O_2_ for 45 min in the dark to block endogenous peroxidase activity. Afterwards, tissue sections were rinsed and preincubated with TBS/BSA 4% for 20 min to avoid unspecific background staining. Incubation with monoclonal primary antibodies of NOX4 (Abcam, Cambridge, UK) and polyclonal antibody of catalase and SOD (Abcam) in a 1 : 100 working solution of TBS/BSA was performed at 4°C overnight in a humidified chamber. Subsequently, sections were washed in TBS and incubated with a peroxidase-labeled polymer conjugated to a goat anti-rabbit immunoglobulin provided as a ready-to-use solution (Envision; Dako A/S, Glostrup, Denmark) for 30 min in a humidified chamber at room temperature. Antibody complexes were visualized using diaminobenzidine for 10 min resulting in brown staining. Thereafter, the slides were rinsed, counterstained with Mayer's hematoxylin, rinsed again, and covered. Negative controls were prepared by omission of the primary antibodies, as well as both the primary and secondary antibody from the staining procedures using TBS/BSA instead. Finally, tissue sections were analyzed using the Zeiss AxioScop 2 microscope (Carl Zeiss, Jena, Germany).

### 2.8. Statistical Analysis

All experiments were performed in triplicate and mRNA quantification is given as means ± SD. Results were compared by ANOVA; Dunnett's and Tukey-Kramer tests were performed as posttest to calculate whether differences between treated and control groups were significant. A *P* value of 0.05 was considered as significant.

## 3. Results

### 3.1. NOX4

**Figure 1:**
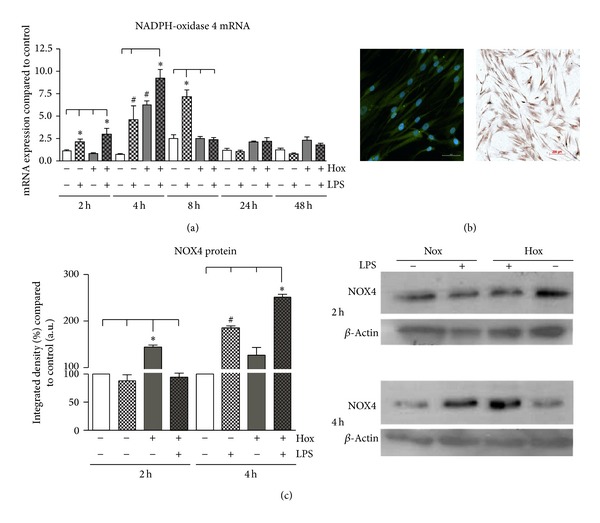
NOX4 in human primary periodontal ligament (PDL) cells. (a) PDL cells were cultured under normoxic or hypoxic condition and stimulated with or without LPS of* Porphyromonas gingivalis* (1 *μ*g/mL). NOX4 mRNA expression was analyzed after 2, 4, 8, 24, and 48 h. Statistical differences were analyzed by one-way ANOVA and post hoc Dunnett and Tukey's multiple comparison test; ^#^ *P* < 0.05 difference to control; ∗*P* < 0.05 difference between groups (means ± SD; *n* = 9). (b) NOX4 protein was visualized by immunofluorescence as well as immunohistochemical staining in unstimulated PDL fibroblasts (1 : 50; antibody from Abcam). (c) Immunoblot data of NOX4 in PDL cells cultured under normoxic or hypoxic condition (Hox) and stimulated with or without LPS of* Porphyromonas gingivalis* (1 *μ*g/mL). NOX4 protein levels were analyzed after 2 and 4 h. Statistical analysis of western blot data was performed using the freely available image-processing software ImageJ 1.43 (http://rsb.info.nih.gov/ij/). Statistical differences were analyzed by one-way ANOVA and post hoc Dunnett and Tukey's multiple comparison test; ^#^ *P* < 0.05 difference to control; ∗*P* < 0.05 difference between groups (means ± SD; *n* = 3). The scale bars indicate 200 *μ*m in the bright field survey and 50 *μ*m in the immunofluorescence image, respectively.

Human primary PDL cells were incubated under normoxic and hypoxic conditions in the presence or absence of LPS-PG (1 *μ*g/mL). Treatment with LPS-PG induced time-dependently a significant increase in the mRNA expression of NOX4 compared to the untreated control (Figure 1(a)). In detail, NOX4 mRNA levels were markedly increased after two hours of LPS-PG stimulation; the increase reached significant levels (4-fold) after 4 hours (*P* < 0.001). NOX4 mRNA levels peaked after 8-hour stimulation with LPS-PG and returned to base line levels afterwards.

Hypoxia alone induced the expression of NOX4 in PDL cells, but in contrast to the stimulation with LPS-PG, the maximum in NOX4 mRNA upregulation was detected after 4 hours (*P* < 0.001; Figure 1(a)). Subsequently, NOX4 mRNA expression decreased; however, it remained at a higher level compared to the control group even after 24 or 48 hours (Figure 1(a)). The highest increase in NOX4 mRNA expression was detected after the combination of both stimuli (8.5 times) which was statistically significant compared to control and even compared to LPS stimulation under normoxia (*P* < 0.001; Figure 1(a)).

To prove whether the increase in mRNA expression was followed by an increase in protein content, immunofluorescence stainings for NOX4 were performed. NOX4 was detectable predominantly in the cytosol and to a slight extent in the nucleus of unstimulated PDL cells (Figure 1(b)). To get more detailed information about the time course of NOX4 protein induction after hypoxic and inflammatory stimulation, we performed immunoblots. After 2 hours only hypoxia provoked a significant increase in NOX4 protein (*P* < 0.001) which diminished afterwards (Figure 1(c)). Under normoxic condition the treatment with LPS-PG induced a significant elevation of NOX4 protein after 4 hours of incubation compared to control and hypoxia alone (*P* < 0.001; Figure 1(c)). The LPS-PG effect was even more pronounced under hypoxic condition which was statistically significant compared to all the other groups (*P* < 0.001; Figure 1(c)). After 24 hours of incubation, alterations of NOX4 protein levels were no longer detectable (data not shown). These findings were in line with our mRNA results even though we detected time-dependent differences.

### 3.2. H_2_O_2_ Formation

**Figure 2:**
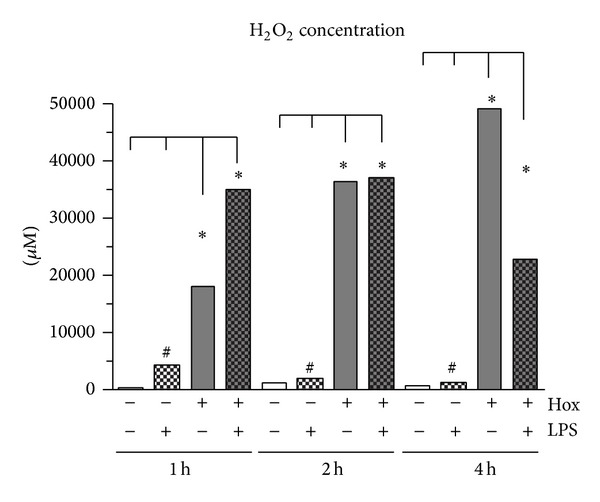
Determination of ROS formation (H_2_O_2_) using Amplex Red assay. PDL cells were cultured under normoxic (Nox) or hypoxic condition (Hox) and stimulated with or without LPS of* Porphyromonas gingivalis* (1 *μ*g/mL). H_2_O_2_ was analyzed after 1, 2 and 4 hours. Statistical differences were analyzed by one-way ANOVA and post hoc Dunnett and Tukey's multiple comparison test; ^#^ *P* < 0.05 difference to control; ∗*P* < 0.05 difference between groups (means ± SD; *n* = 6).

To determine hydrogen peroxide generation, we used the commercially available Amplex Red-horseradish peroxidase assay. PDL cells displayed a low constitutive H_2_O_2_ production under normoxic conditions. We observed a significant upregulation of the H_2_O_2_ concentration in the cell culture supernatant after 1, 2, and 4 hours of stimulation with LPS-PG. Hypoxia alone induced a significant increase in H_2_O_2_ formation compared to the time-matched controls (Figure 2). While in the LPS group the H_2_O_2_ values were the highest after 1 hour and decreased continuously after 2 and 4 hours (Figure 2), under hypoxic condition H_2_O_2_ increased permanently (Figure 2). Comparing the LPS and the hypoxic effect revealed that hypoxia alone induced a significantly higher H_2_O_2_ accumulation over the complete observation period (*P* < 0.001). However, the combination of both stimuli elicited the highest H_2_O_2_ concentration after 1 hour which was significant not only compared to control but also compared to the LPS and the hypoxia group (Figure 2). After 2 hours the treatment with LPS-PG was no longer able to enhance the hypoxia-induced H_2_O_2_ formation; it was still significantly increased compared to control and the LPS stimulation but no longer compared to the hypoxic group. This was even more pronounced after 4 hours.

### 3.3. Redox Systems

**Figure 3:**
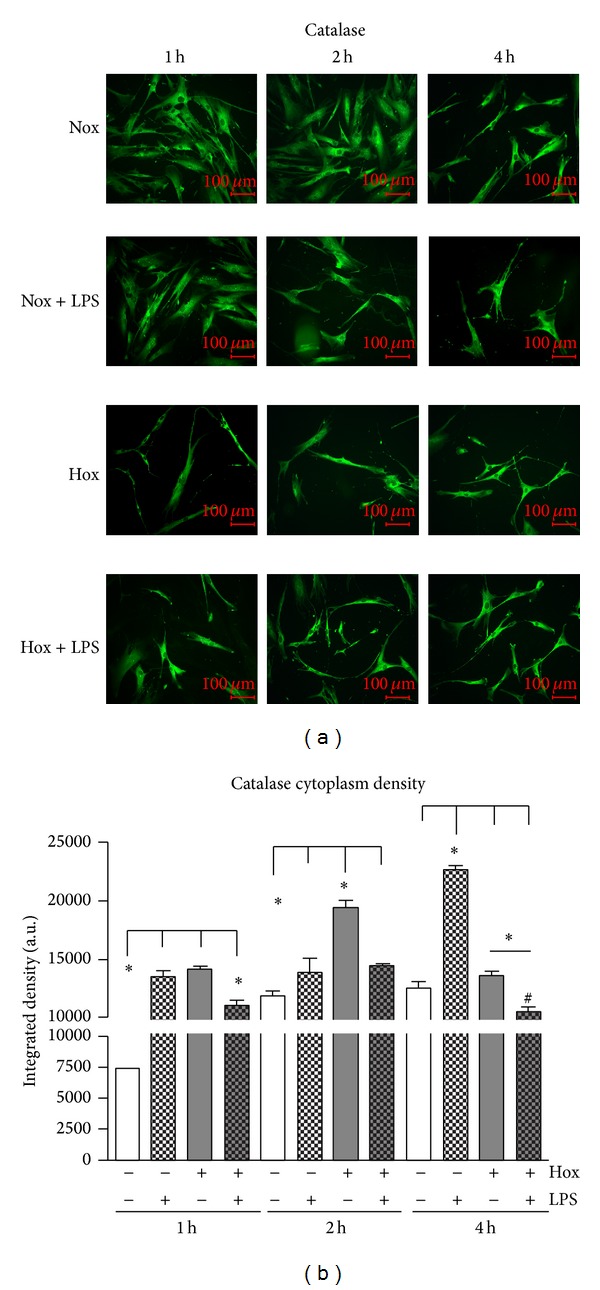
Catalase (CAT) in PDL cells. (a) CAT was visualized by immunofluorescence staining (1 : 50; antibody from Abcam). PDL cells were cultured under normoxic (Nox) or hypoxic condition (Hox) and stimulated with or without LPS of* Porphyromonas gingivalis* (1 *μ*g/mL). Catalase cytoplasm density (b) was determined in relation to cell area using the freely available image-processing software ImageJ 1.43 (http://rsb.info.nih.gov/ij/). Statistical analysis of immunofluorescence data was analyzed by one-way ANOVA and post hoc Dunnett and Tukey's multiple comparison test; ^#^ *P* < 0.05 difference to control; ∗*P* < 0.05 difference between groups (means ± SD; *n* = 6). The scale bars indicate 100 *μ*m in the immunofluorescence images.

**Figure 4:**
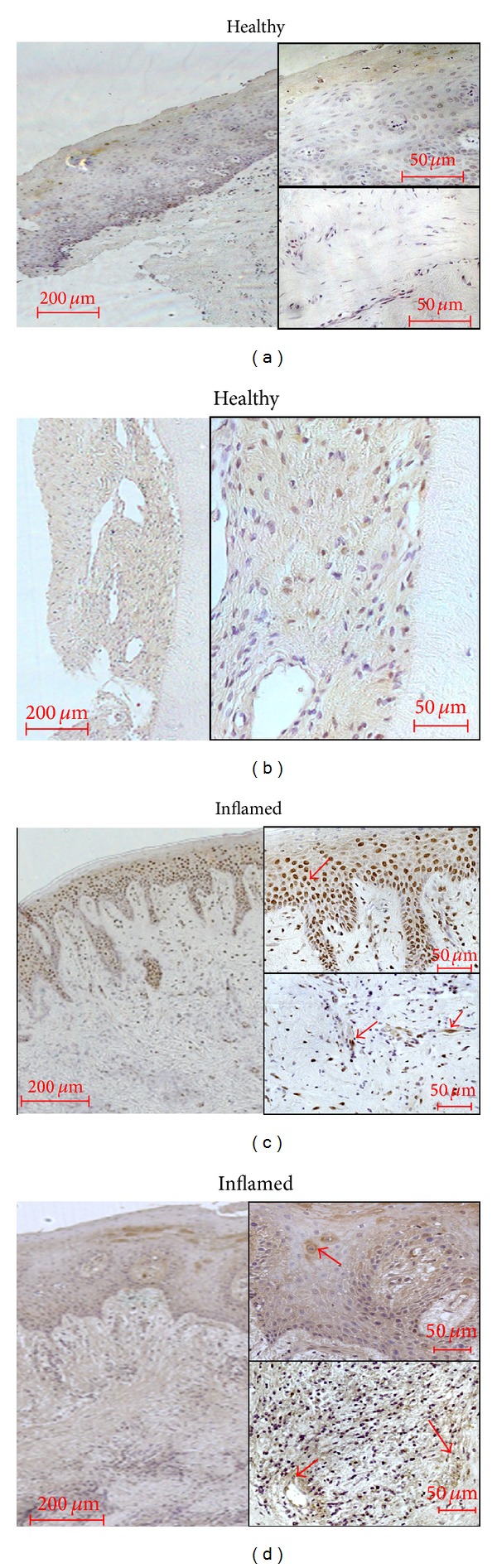
NADPH-oxidase 4 (NOX4) occurrence in healthy and inflamed human tissue. Healthy gingival epithelium (a), periodontal ligament (b), and samples of gingivitis (c) or periodontitis (d) were obtained after the approval of the Ethics Committee of the University of Bonn and parental as well as patient's allowance (*n* = 3). Monoclonal primary antibody against NOX4 (Abcam, Cambridge, UK) was used in a concentration of 1 : 100. In gingival and PDL fibroblasts as well as in immune cells like leukocytes and in vessels, the immunoreactivity was primarily restricted to the cytosol and cell walls. Red arrows indicate examples for the typical distribution (Figures 4(c) and 4(d)). The scale bars indicate 200 *μ*m in the surveys and 50 *μ*m in the higher magnifications, respectively.

**Figure 5:**
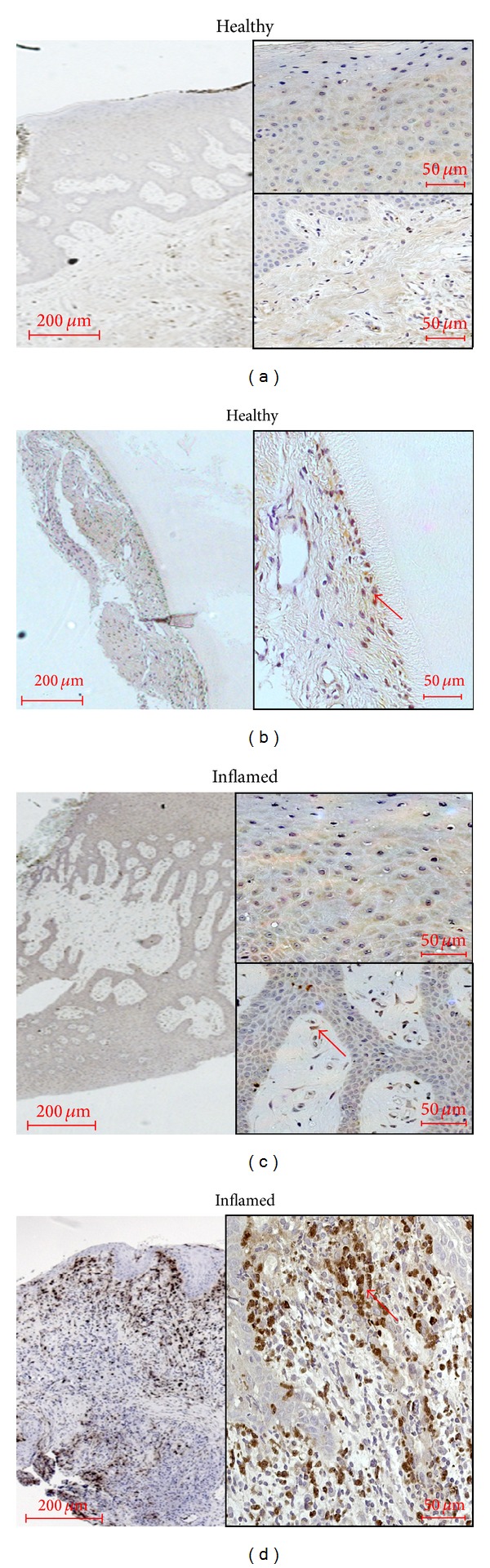
Catalase (CAT) occurrence in healthy and inflamed human tissue. Healthy gingival epithelium (a), periodontal ligament (b), and samples of gingivitis (c) or periodontitis (d) were obtained after the approval of the Ethics Committee of the University of Bonn and parental as well as patient's allowance (*n* = 3). Polyclonal primary antibody against catalase (Abcam, Cambridge, UK) was used in a concentration of 1 : 100. No immunostaining could be found in the healthy gingival epithelium. In the healthy PDL, predominantly PDL cells located near to the tooth root surfaces were stained (Figure 5(b), red arrow). In gingivitis tissue, a weak staining of gingival keratinocytes similar to healthy tissue could be seen (Figure 5(c)). Instead, in the tissue samples of patients with periodontitis a strong immunoreactivity was observed in the subepithelial layer probably referring to nuclei staining of immune cells like leukocytes. Red arrows indicate examples for the typical localisation (Figure 5(d)). The scale bars indicate 200 *μ*m in the surveys and 50 *μ*m in the higher magnifications, respectively.

**Figure 6:**
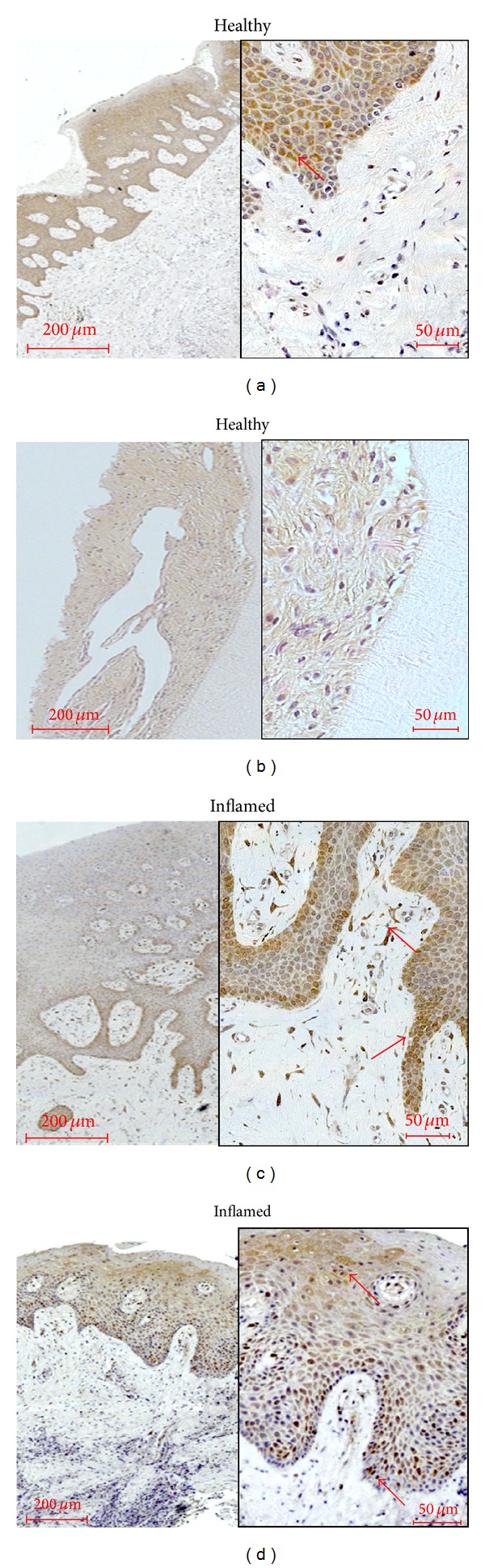
Superoxide dismutase (SOD) occurrence in healthy and inflamed human tissue. Healthy gingival epithelium (a), periodontal ligament (b), and samples of gingivitis (c) or periodontitis (d) were obtained after the approval of the Ethics Committee of the University of Bonn and parental as well as patient's allowance (*n* = 3). Polyclonal primary antibody against SOD (Abcam, Cambridge, UK) was used in a concentration of 1 : 100. Gingival epithelium showed a moderate to strong staining of SOD in keratinocytes which was primarily limited to the cytosol (Figure 6(a) and red arrow depicts the typical localization). In the healthy periodontium, gingival and PDL fibroblasts were weakly stained (Figure 6(b)). In inflamed tissues, basal epithelial cells seemed to be stained more intensively in the gingivitis specimen than in periodontitis (Figures 6(c) and 6(d)). In addition, the subepithelium revealed immunoreactivity in local cells like gingival fibroblasts and scattered immune cells (Figure 6(c), see red arrow). In periodontitis, SOD immunostaining seemed to be limited to the gingival epithelium (Figure 6(d); see red arrow). The scale bars indicate 200 *μ*m in the surveys and 50 *μ*m in the higher magnifications, respectively.

To analyze which protective mechanisms against ROS formation are involved in our setting, we determined the redox system components catalase (CAT) and superoxide dismutase (SOD) in PDL cells after treatment with LPS-PG under normoxic and hypoxic conditions by immunofluorescence (Figures 3(a)-3(b)). In addition, periodontal tissues from clinically healthy patients (I) as well as from patients with the diagnosis of gingivitis (II) and periodontitis (III) were obtained from three different patients for each group and stained immunohistochemically for NOX4, CAT, and SOD (Figures 4, 5, and 6).

#### 3.3.1. Immunofluorescence of Redox Systems in PDL Cells

PDL cells displayed a constitutive cytosolic catalase expression under normoxic conditions. Treatment with LPS-PG and hypoxia induced a change in PDL cell morphology, which was first obvious after a 1-hour incubation under hypoxic conditions and after 2 hours with LPS-PG (Figure 3(a)). As shown in Figure 3(b), analyzing catalase cytoplasm density in relation to constant cell area revealed a significant upregulation of catalase in PDL cells stimulated with LPS-PG or hypoxia after 1 h (*P* < 0.001; Figure 3(b)). This was also true for hypoxic conditions after 2 h compared to all other groups. Under inflammatory stimulation (LPS) a significant increase of catalase cytoplasm density was detectable after 4 hours compared to all the other groups (*P* < 0.001; Figure 3(b)). Instead, the combination of both stimuli diminished the hypoxic, as well as LPS-induced effects after 1, 2, and 4 hours. Determining superoxide dismutase protein expression in PDL cells showed no significant differences at any time point (*P* > 0.05; data not shown).

#### 3.3.2. Immunohistochemistry of NOX4 and Redox Systems in Periodontal Tissue Sections

Periodontal tissues (healthy gingiva and periodontal ligament (PDL), gingivitis, and periodontitis) were obtained from three different patients for each group.

*NOX4.* In the healthy periodontal tissue samples only very weak immunostaining for NOX4 could be obtained (Figures 4(a) and 4(b)). In the PDL weak immunoreactivity with a moderate staining of some fibroblasts was observed. In inflamed tissues from patients suffering from gingivitis or periodontitis, strong immunoreactivity for NOX4 appeared in the cytosol and even stronger in the nuclei of keratinocytes of the basal layer of inflamed gingival epithelium (Figures 4(c)-4(d)). In gingival and PDL fibroblasts as well as in immune cells like leukocytes and in vessels, the immunoreactivity was primarily restricted to the cytosol and cell walls (Figures 4(c)-4(d); see red arrows). 

*CAT. *Nearly no immunostaining could be found in the healthy gingival epithelium (Figure 5(a)). In the healthy PDL, predominantly PDL cells located near to the tooth root surfaces were stained (Figure 5(b); see red arrow). In gingivitis tissue, a weak staining of gingival keratinocytes similar to healthy tissue could be seen (Figure 5(c)). Instead, in the tissue samples of patients with periodontitis a strong immunoreactivity was observed in the subepithelial layer probably referring to nuclei staining of immune cells like leukocytes (Figure 5(d); see red arrows).

*SOD.* As for healthy tissues, gingival epithelium showed a moderate to strong immunoreactivity of keratinocytes which was primarily limited to the cytosol (Figure 6(a); see red arrow). In healthy periodontium, gingival and PDL fibroblasts were weakly stained (Figure 6(b)). Compared to inflamed tissues, basal epithelial cells seemed to be stained more intensively in the gingivitis specimen (Figures 6(c)-6(d)). In addition, the subepithelium revealed immunoreactivity in local cells like gingival fibroblasts and scattered immune cells (Figure 6(c)). In periodontitis stained sections, SOD immunostaining seemed to be limited to the gingival epithelium (Figure 6(d); see red arrows). In inflamed tissues subepithelial layers showed no signs of immunoreactivity.

## 4. Discussion

Oxidative stress is discussed as an important cofactor in the etiology and pathogenesis of several oral and dental diseases, for example, in inflammatory processes like periodontitis. First evidence for the presence and role of ROS in the periodontium was given by the detection of these molecules after respiratory burst by polymorphonuclear lymphocytes (PMNLs) in periodontal damage [22]. Since then, the implication of ROS and their degrading enzymes SOD or CAT in the pathogenesis of periodontal diseases has been supported by several groups [23–26]. To analyse whether cellular redox systems in the periodontium are affected by inflammatory conditions, we investigated the effect of hypoxia and LPS from* Porphyromonas gingivalis* (LPS-PG) on periodontal ligament cells. We showed that both stimuli induced a significant increase of NOX4 and a significant upregulation of hydrogen peroxide generation. NOX enzymes were first described in phagocytes as being responsible for defense mechanisms against pathogens using NOX-generated ROS molecules. Till then, NOX4 expression has been detected in nonphagocyte cell types like kidney cells, osteoclasts, and osteoblasts as well as dermal cells [9, 11, 47]. In PDL cells, hypoxia and LPS-PG induced a significant increase of NOX4 mRNA. The protein expression was mainly located in the cytosol but likewise in the nucleus. Our findings were in line with Brown and Griendling who have shown that the typically transmembrane molecule is localized in different cell compartments [9].

NOX4 is one of the major sources of ROS generation which is highly and ubiquitously expressed compared to the other family members. Mandal and colleagues demonstrated that apart from its antimicrobial function NOX4 is responsible not only for osteoclast but also for preosteoblast differentiation via ROS accumulation reinforcing the idea of NOX4 as a key regulator in bone remodeling under physiological ROS amounts [47]. Animal studies support the hypothesis that massive ROS formation induced bone resorption via NOX4 [20, 21]. Whereas physiological levels of NOX4-generated ROS in PDL cells might facilitate alveolar bone remodeling, massive ROS accumulation during inflammatory periodontal diseases might lead to alveolar bone destruction favoring tooth loss. We have demonstrated first that NOX4 is upregulated in periodontal ligament fibroblasts during bacterial and hypoxic events. Hence, PDL fibroblasts may be a source of excessive ROS generation and are therefore of particular importance during periodontal diseases.

Our results support current findings determining the role of oxidative stress in different periodontal pathologies which implicate that the balance between NADPH-oxidases and redox systems is crucial to maintain cell and tissue homeostasis [22–26]. However, there are only a few studies using human periodontal fibroblast in the context of oxidative stress and none of them evaluated the role of NOX4 and counteracting redox systems during inflammatory conditions with LPS-PG stimulation and hypoxia. An increase of ROS generation was detected by San Miguel et al. following PDL fibroblast stimulation with nicotine or ethanol which were abolished after antioxidant treatment [40]. They completed their results using metal ions like nickel for PDL stimulation which increases ROS activity in these cells indicating a role in metal cytotoxicity or even nickel allergy [41]. Transient metal ions like nickel and cobalt are known to mimic cellular hypoxia mainly due to the inhibition of prolyl hydrolases (PDHs) and asparaginyl hydrolase (factor-inhibiting HIF (FIH)). The inhibition of hydroxylation leads to HIF-1*α* nuclear translocation followed by the transcription of diverse genes which are implicated in hypoxic adaptation, immune response, or angiogenesis, therefore indirectly supporting our results under hypoxic condition [48, 49].

We could show that hypoxia as well as LPS-PG induced ROS formation by detecting H_2_O_2_ using the commercially available Amplex Red assay. The high levels of H_2_O_2_ released under hypoxic conditions after 1 and 2 hours in PDL cells may be caused by different mechanisms. First, we found a significant upregulation of the mRNA and protein of the H_2_O_2_-generating enzyme NOX4. Second, the lack of molecular oxygen as the final electron acceptor in the respiratory chain may be responsible for the early release of ROS. LPS-PG alone induced acutely a small but significant increase in H_2_O_2_ release and enhanced the hypoxia-induced generation. In human gingival fibroblasts similar results were obtained, showing the increase of ROS and antioxidants after* P. gingivalis* or LPS stimulation [26, 42, 43]. Interestingly, despite the increase in NOX4 mRNA and protein expression after 4 hours of stimulation with LPS-PG under hypoxic conditions, the H_2_O_2_ generation was significantly reduced when compared to hypoxia alone. An additional inhibition of the respiratory chain by LPS-PG which was described for cardiac mitochondria [50] is therefore conceivable.

To antagonize oxidative stress, cells developed several antioxidative enzyme systems. Therefore, we measured SOD and CAT expression in PDL cells and periodontal tissues by immunofluorescence and immunohistochemistry. SODs are metalloenzymes which are responsible for the dismutation of superoxide radicals to hydrogen peroxide [51]. The reduction of hydrogen peroxide to water and oxygen is subsequently catalyzed by CATs which are ubiquitously expressed. Both enzymes are essential to maintain cellular redox homeostasis. The present data imply that PDL cells and periodontal tissues have fundamental defense machineries against bacteria represented by NOX4-dependent inducible ROS generation as well as protective redox systems. This is of great importance concerning the high amounts and diversity of the oral flora. Different studies indicate that redox systems are key players in the development of periodontal diseases and markers for periodontal health status [52–56]. We detected a significant increase of catalase in PDL cells in response to LPS as well as hypoxia after 1 and 2 hours. In our setting, PDL fibroblasts seem to counteracted LPS- and hypoxia-induced ROS formation with the elevation of CAT expression. But prolonged incubation under hypoxic conditions failed to increase catalase. Since ROS generation was still elevated at these later time points, a delay in ROS degradation may be the explanation. Taken together, the dramatic elevation in ROS formation by the combination of hypoxia and LPS-PG on one hand and the reduction of catalase under the same conditions on the other hand increase the oxidative stress in the periodontium favoring the progression of inflammation and bone resorption and finally may lead to tooth loss.

The fundamental role of the balance between ROS generation and antioxidative mechanisms in periodontal inflammatory processes was supported by our immunohistochemical findings. In patients suffering from periodontal inflammatory diseases like gingivitis and periodontitis, we demonstrated that NOX4 is upregulated and thus likely responsible for H_2_O_2_ generation. However, we detected as well an increase in catalase abundance in inflamed gingival and periodontal tissue which was in line with data from Krifka and colleagues who detected high CAT levels in monomer-exposed macrophages as a response to oxidative stress [57]. In our* in vitro* setting, PDL fibroblasts seem to counteract LPS- and hypoxia-induced ROS formation predominantly with an elevation of CAT expression since we could not detect any SOD immunoreactivity. The role and impact of the antioxidative systems SOD and CAT are controversially discussed. Recent studies measured CAT and SOD activities in gingival tissues of patients with periodontitis [58, 59]. Borges Jr. et al. did not find differences in CAT activities comparing healthy and diseased patients [58]. Instead, Tonguç et al. detected decreased local SOD and CAT activities in periodontitis patients which seemed to be increased by smoking [59]. In contrast, in inflamed tissues we observed high amounts of SOD which was localized primarily in epithelial cells. Jacoby and Davis determined SOD in periodontal soft tissues. They detected twice as much SOD activity in the periodontal ligament as in the human skin (dermis) [60]. Furthermore, immunohistochemical analysis revealed that SOD is localized in the periphery of matrix collagen fibrils and to the glycocalyx of tissue fibroblasts. This was confirmed by Skaleri$\overset{ˇ}{\text{c}}$ et al. [42] which might explain the fact that we did not detect significant differences of SOD levels after hypoxic and LPS-PG stimulation intracellularly in PDL fibroblasts. Another reason for these results may be caused by the age of the PDL cells used in this study. We explanted the PDL cells from young and oral healthy patients but most of the immunohistochemical stainings were performed in tissues from elderly patients with clearly diagnosed gingivitis or periodontitis. Therefore it is possible that in PDL cells from elderly patients a different expression pattern of antioxidant enzyme systems could be found which further deteriorate the redox state of the periodontal ligament. Moreover, other authors found reduced antioxidants in the body fluids of patients with periodontitis indicating a dysregulation which would favor disease progression [23, 24, 61]. A variety of* in vitro* and animal studies analyzed the effect of antioxidant supplement in the context of inflammatory or cytotoxic events [57, 62, 63]. They have shown that antioxidants inhibit these processes indicating a therapeutic approach in pathologies like periodontitis. First clinical investigations confirmed the efficiency of antioxidant micronutrients and adjuncts in periodontal diseases [22, 54, 56, 64, 65]. The present data indirectly support these studies.

Concerning periodontal pathogen invasiveness and virulence, there is compelling evidence that* P. gingivalis* possesses its own protective antioxidants, for example, rubrerythrin defending them against the oxidative burst of the host [66]. We have shown that its virulence factor LPS-PG induced high amounts of ROS via NOX4 which may result in bone resorption favoring the invasiveness of the pathogen which is at the same time protected by its own defense machinery. This would further explain the pathogenicity and resistance of this bacterium strain in patients with periodontitis.

## 5. Conclusion

In this study we have demonstrated that LPS from* Porphyromonas gingivalis* (LPS-PG) as well as hypoxia induces a NOX4-dependent increase in H_2_O_2_ release in PDL fibroblasts which may contribute to the development and progression of periodontal diseases when not balanced by a concomitant increase in antioxidative systems. The dramatic elevation in ROS formation induced by the combination of hypoxia and LPS-PG and the reduction of catalase under the same conditions increased the oxidative stress in the periodontium favoring the progression of inflammation, bone resorption, and possibly tooth loss.

Taken together, the presented data further explain the destructive mechanism of hypoxic and inflammatory conditions in the periodontium and may improve diagnostic and therapeutic strategies in periodontal diseases.
